# Intestinal injury and gut permeability in sickle cell disease

**DOI:** 10.1186/s12967-019-1938-8

**Published:** 2019-05-30

**Authors:** Dibyendu Dutta, Barbara Methe, Salomon Amar, Alison Morris, Seah H. Lim

**Affiliations:** 1 Division of Hematology and Hemostasis, New York Medical College, 19 Bradhurst Avenue, Suite 2575S, Hawthorne, Valhalla, NY 10595 USA; 2 Center for Medicine and the Microbiome, Department of Medicine, University of Pittsburgh, Pittsburgh, PA 15213 USA; 3 Department of Immunology and Microbiology, New York Medical College, Valhalla, NY 10595 USA

**Keywords:** Intestinal injury, Gut permeability, Sickle cell disease

## Abstract

**Background:**

Due to recurrent hypoxia-reperfusion injury induced by vaso-occlusive crises (VOC), patients with sickle cell disease (SCD) may have intestinal injury and increased permeability. These may explain the qualitative and quantitative neutrophil abnormalities observed in these patients.

**Methods:**

Serum intestinal fatty-acid binding protein (iFABP), lipopolysaccharides (LPS), and CD62L were measured by ELISA. Multicolor flow cytometry was used to measure circulating aged neutrophils.

**Results:**

Compared to controls, SCD individuals had higher iFABP (median: 1.38 ng/ml vs 0.81 ng/ml; *p *= 0.04) and LPS (median: 2.15 μg/ml vs 0.69 μg/ml; *p* = 0.03), indicating intestinal injury and increased intestinal bacterial translocation into the systemic circulation. They also had higher soluble CD62L (median: 1.38 μg/ml vs 1.11 μg/ml; *p* = 0.04). Among SCD individuals, soluble CD62L correlated positively with circulating aged neutrophils (*R *= 0.7, *p* = 0.03) and LPS (*R* = 0.66, *p* = 0.027). Surprisingly, serum iFABP in SCD correlated negatively with both LPS (*R* = − 0.7, *p* = 0.02) and soluble CD62L (*R* = − 0.56, *p* = 0.08).

**Conclusions:**

Since LPS translocation across the intestinal barrier may be due to increases in the intestinal bacterial density, gut permeability, or both, the negative correlations between iFABP and LPS, and CD62L raise the possibility that any damage-associated molecular patterns induced by intestinal injury may modulate the degree of bacterial translocation. Our results provide the first evidence of the presence of intestinal injury and increased gut permeability in SCD.

## Background

SCD is associated with progressive multi-organ dysfunction. Although the sequelae of SCD are well-described in the pulmonary, neurologic, renal, and cardiovascular systems, evidence implicating abnormalities in the gastrointestinal system is lacking. Indirect evidence supporting VOC affecting the intestine includes the reported cases of ischemic colitis in SCD [1–3]. The propensity for the splenic artery, part of the splanchnic vasculature, of pediatric SCD to develop atherosclerosis [4] also supports VOC occurring in the intestinal vasculature. However, direct evidence implicating changes in the intestinal pathophysiology is lacking.

## Main text

We and others have recently provided the first direct evidence of abnormalities in the intestine in SCD. We demonstrated that the intestinal microbiome is altered in these individuals [5, 6]. The altered intestinal microbiome is most likely the result of VOC causing hypoxia-reperfusion injury [7]. Probably as a result of a compensatory increase in abundance of intestinal *Clostridiales*, the altered intestinal microbiome appeared to protect SCD individuals from *Clostridium difficile* infection (CDI) compared to hospital-wide populations [8]. To further evaluate how the intestine might be affected by SCD, we have set out in this study to identify evidence for intestinal injury and gut permeability and the consequences of these pathologic changes in these individuals.

## Materials and methods

### Subjects and specimens

Subjects with Hb SS (n = 11) and iron deficiency anemia (n = 8) of comparable hemoglobin were included in the study. SCD subjects were included in the study only if they had not developed any painful VOC within the 4 weeks of the specimens being collected. We chose individuals with iron deficiency anemia but without SCD as controls to exclude any contribution anemia per se might have on the measured parameters. The smoking habit, gender distribution, age, and weight between the two groups were also comparable. The study was approved by the Institution Review Board.

### Analysis of circulating aged neutrophils

Peripheral blood was lysed with ACK lysing buffer (Gibco, Cat# A1049201) and the nucleated cells were stained with the following conjugated monoclonal antibodies: CD62L-APC, CD115-PE-Cy7, CXCR4-PE, and Gr-1-FITC (all from eBioscience, USA). Flow cytometry was carried out on the Moflo XDP Sorter (BD Biosciences). Dead cells were excluded by FSC, SSC and Propidium iodide. Neutrophils were gated by Gr-1^hi^ CD115^lo^ SSC^hi^ and aged neutrophils gated by CD62L^lo^ CXCR4^hi^ within the neutrophil population.

### Measurements of soluble CD62L, LPS, and iFABP

Peripheral markers of intestinal integrity were measured by enzyme-linked immunosorbent assays using commercially available kits: iFABP (Life Technologies, USA), soluble CD62L (Life Technologies, USA) and LPS (Cloud-Clone Corp. USA). All measurements were made in triplicates and the results confirmed in one independent repeat of the experiment.

## Results and discussion

**Fig. 1:**
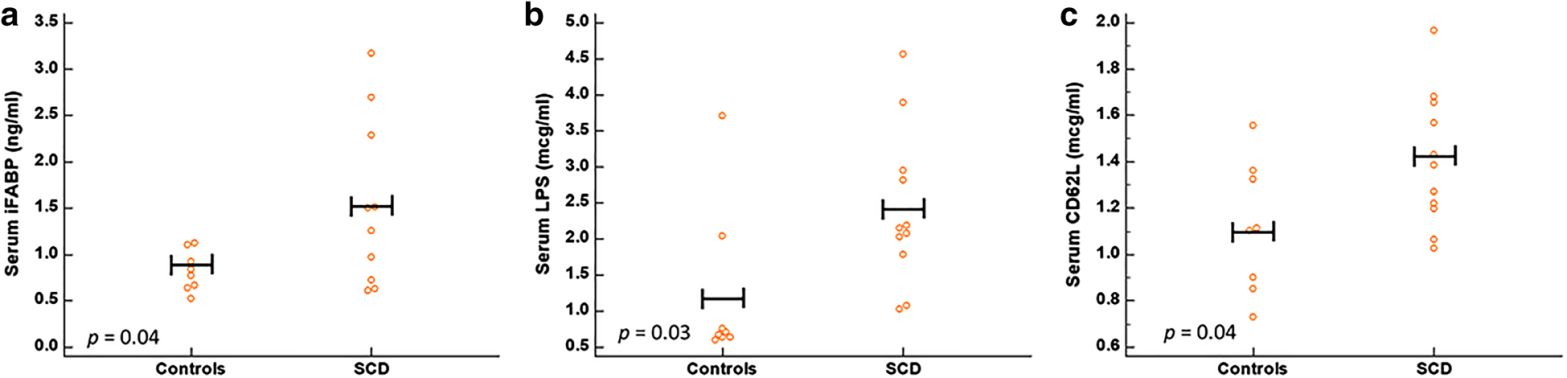
Comparison between individuals with sickle cell disease (SCD) and controls with iron deficiency anemia of comparable hemoglobin. **a** SCD patients had higher levels of serum iFABP compared to controls, indicating the presence of intestinal injury in SCD. **b** SCD patients had higher levels of serum LPS compared to controls, indicating increased amounts of bacterial products being translocated across SCD intestinal barrier to the systemic circulation. **c** SCD patients had higher levels of soluble CD62L compared to controls, indicating increased peripheral neutrophil activations in SCD. (Horizontal bars represent the means)

Hypoxia-reperfusion injury causes tissue damage. Intestinal damage would, therefore, be expected if sickle cell VOC affects the splanchnic vasculature. Intestinal FABP is expressed in enterocytes of the small intestine. Since iFABP is released into the systemic circulation when there is intestinal damage, measurements of the serum iFABP in SCD would provide the evidence for intestinal injury due to VOC affecting the splanchnic vasculature. In this study, the serum iFABP in SCD individuals was significantly higher than that in the control group (median: 1.38 ng/ml [range 0.61–3.17] vs 0.8 ng/ml [range 0.53–1.12]; two-tailed *p* = 0.04) (Fig. 1a), providing the first evidence of intestinal injury in SCD. In addition, we found increased translocation of bacterial products across the intestinal barrier into the systemic circulation in SCD, as measured by the higher serum LPS levels (median: 2.15 μg/ml [range 1.03–4.56] vs 1.2 μg/ml [range 0.60–3.70]; two-tailed p = 0.03) (Fig. 1b). In keeping with the findings in a previous study [9], SCD individuals in our study also had higher levels of soluble CD62L (median: 1.38 μg/ml [range 1.03–1.97] vs 1.11 μg/ml [0.73–1.55]; two-tailed *p* = 0.04) (Fig. 1c), indicating the presence of higher activated neutrophils in circulation.

**Fig. 2:**
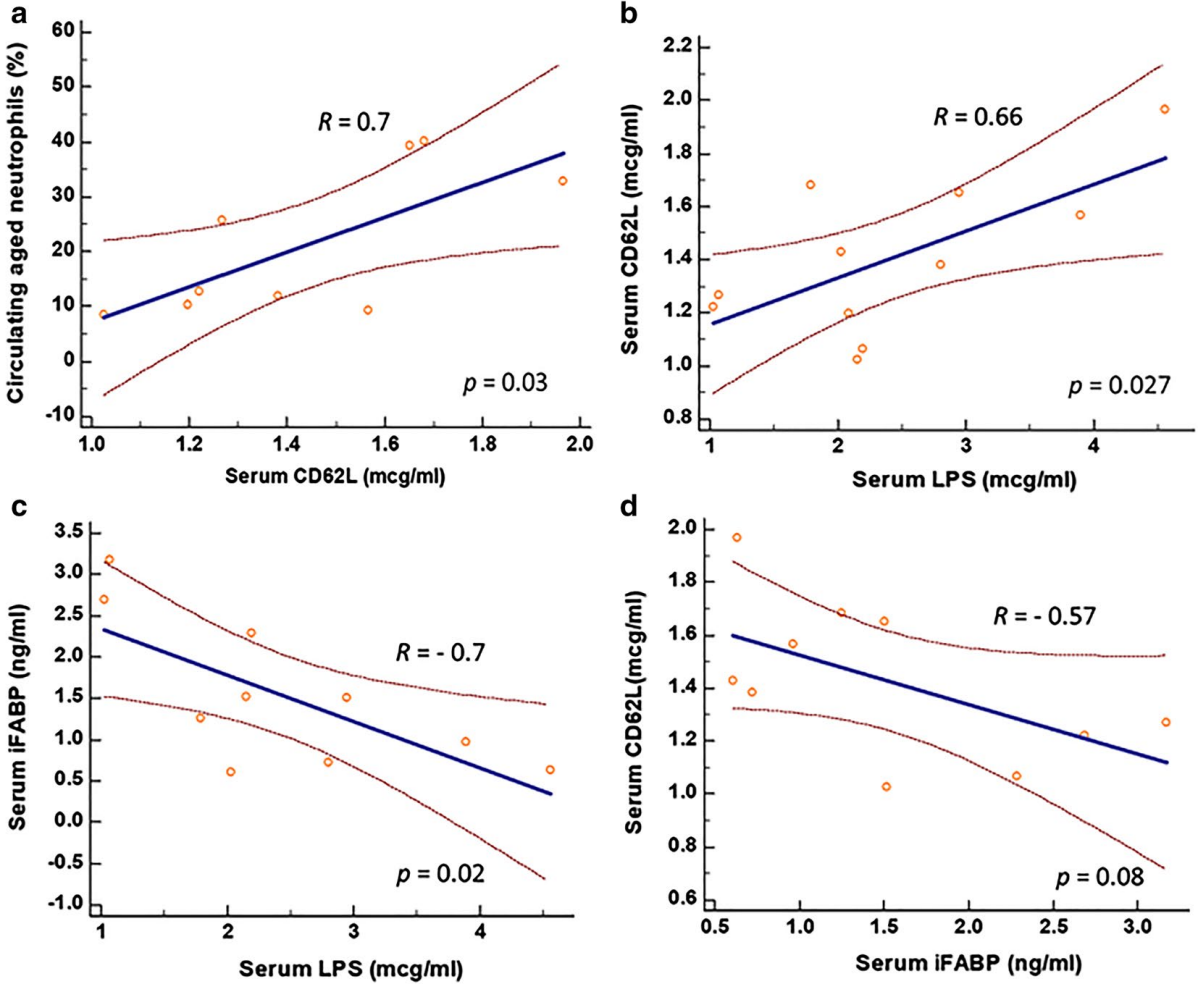
Correlations between soluble CD62L and predetermined parameters in SCD individuals. **a** Soluble CD62L correlated closely with percent circulating aged neutrophils, supporting the utilization of CD62L as the surrogate for circulating aged neutrophils in our subsequent experiments. **b** Serum LPS correlated positively with soluble CD62L, suggesting the role of LPS in modulating circulating aged neutrophils. **c** iFABP correlated negatively with serum LPS, and **d** with soluble CD62L in SCD, raising the possibilities that either DAMPs induced by intestinal injury modulate intestinal microbial density, or rapid cell turnover of enterocytes induced by intestinal injury produces new enterocytes well-endowed with functional tight junctions that reduce bacterial product translocation across the intestinal barrier

Aged neutrophils are pivotal in the pathogenesis of VOC. Since we found, in SCD, that soluble CD62L was elevated, we next determined how soluble CD62L correlated with percent circulating aged neutrophils. Elevated serum soluble CD62L predicted for higher percent of circulating aged neutrophils (*R* = 0.7; two-tailed *p* = 0.03) (Fig. 2a). This result supports the utilization of soluble CD62L as the surrogate for circulating aged neutrophils in our subsequent data analysis. We identified that serum LPS correlated positively with soluble CD62L (*R* = 0.66; two-tailed *p* = 0.027) (Fig. 2b), supporting the notion that LPS translocated across the intestinal barrier into the systemic circulating might be, at least in part, responsible for modulating circulating aged neutrophils in SCD.

Since elevated translocation of LPS across the intestinal barrier may be the result of increase in either gut permeability or intestinal microbial density, we next determined the relationship between serum iFABP and LPS. If serum LPS is solely due to a compromised gut barrier, serum iFABP in SCD would be expected to correlate positively with LPS levels and percent of circulating aged neutrophils. However, to our surprise, a negative correlation between serum LPS and iFABP (*R* = − 0.7; two-tailed *p* = 0.02) (Fig. 2c) was observed. Similarly, a negative correlation trending towards significance occurred between serum iFABP and soluble CD62L (*R* = − 0.57; two-tailed *p* = 0.08) (Fig. 2d). These negative correlations may be accounted for by one of two explanations, or both. First, in SCD individuals with elevated iFABP reflecting a more intense degree of intestinal injury, high levels of damage-associated molecular patterns (DAMPs) are produced that are bactericidal, leading to reduction in the intestinal microbial density that in turns decrease the LPS available for translocation across the gut barrier. Second, SCD individuals with elevated iFABP reflecting a more intense degree of intestinal injury are associated with a more rapid cell turnover of enterocytes, producing new enterocytes that are endowed with healthy tight junctions to reduce the gut permeability and LPS translocation.

SCD individuals have higher white cell counts than those with Hb AA phenotype [10]. Their neutrophils showed higher levels of activation molecules, e.g. CD64 [9] and CD11b/CD18 [11], and they have elevated soluble CD62L, a marker of neutrophil activation [9]. In SCD mice, sickled erythrocytes were more likely to adhere to activated neutrophils than to endothelium [12]. Immobilized neutrophils are the niduses for sickled erythrocytes to attach to and cause VOC. SCD mice treated with broad-spectrum antibiotics had lower number of circulating aged neutrophils and were protected from fatal tumor-necrosis factor-α (TNFα)-induced VOC [13]. Aged neutrophils are regulated in mice via toll-like receptor (TLR) 2/4 and Myd88 [14], both are receptors for pathogen-associated molecular patterns (PAMPS) [14, 15]. Neutrophils are commonly activated in response to the release of inflammatory cytokines following receptor recognition of PAMPs. We previously postulated that an altered intestinal microbiome or increased microbial density in the setting of a compromised intestinal barrier allows enhanced bacterial translocation into the bloodstream. These microbes or their products encounter and activate neutrophils, potentially explaining the higher baseline neutrophils and circulating aged neutrophils [16]. The results of our current study support this hypothesis.

## Conclusions

In summary, SCD is associated with intestinal injury and increased bacterial translocation across the gut barrier. The degree of bacterial translocation into the systemic circulation is likely determined by the balance between changes in the gut permeability and control of intestinal microbial density by DAMPs produced that might be a result of intestinal injury. Further study into this delicate interaction may provide clues to future therapeutic approaches for the disease.

## Data Availability

Yes, upon request.

## Abbreviations

CDI: *Clostridium difficile* infection
DAMPs: damage-associated molecular patterns
Hb SS: sickle cell hemoglobin
iFABP: intestinal fatty acid binding proteins
LPS: lipopolysaccharides
PAMPs: pathogen-associated molecular patterns
SCD: sickle cell disease
TLR: toll-like receptor
TNF: tumor necrosis factor
VOC: vaso-occlusive disease
